# Editorial: Developmental origins of health and disease: Impact of preterm birth

**DOI:** 10.3389/fped.2022.1120208

**Published:** 2023-01-04

**Authors:** Lynette Kay Rogers, Jonathan L. Slaughter

**Affiliations:** ^1^ Department of Pediatrics, The Ohio State University, Columbus, OH, United States; ^2^ Department of Pediatrics, Abigail Wexner Research Institute, Nationwide Children’s Hospital, Columbus, OH, United States

**Keywords:** preterm birth, maternal environment, intrauterine growth restriction, adult disease, prematurity

**Editorial on the Research Topic** Developmental origins of health and disease: Impact of preterm birth

The pivotal concept of developmental origins of disease was first identified in the 1980s and has been the topic of numerous investigations related to adult-onset diseases such as obesity, diabetes, and coronary artery disease (1). Maternal-fetal interactions effect the fetus directly in the context of maternal illness or nutritional deprivation or can establish epigenetic changes that effect basic metabolic function such as obesity, diabetes, and the HPA axis (2, 3). While preterm birth itself is not a direct maternal influence, it is often the result of an adverse *in utero* environment that precipitates the early birth. Further, preterm birth has been associated with impaired structural and function development of organ systems in the offspring, thus making preterm birth a unique predisposing factor for adult disease (4). The manuscripts published within this special issue address factors associated with maternal health, growth of the infant, and morbidities associated with early birth and poor growth.

Mautner et al. assessed maternal physical and mental health between women with early preterm birth vs. late preterm birth. Early preterm birth was associated with depressive symptoms and lower health-related quality of life (HRQoL) scores, but late preterm birth mothers had even higher depressive symptoms; both of which may affect the care provided to their respective infants.” Both Motte-Signoret et al. and Gonzales-Garcia et al. studied small for gestational age or intrauterine growth restricted (IUGR) infants. Motte-Signoret measured growth factor (GF), insulin growth factor 1 (IGF1), and insulin resistance in small for gestational age (SGA) infants. SGA infants demonstrated resistance to GF and IGF1 with insulin resistance. These findings could explain initial defects in early catch-up growth, risks for later catch-up growth, and higher prevalence of metabolic syndrome in later life. Gonzales-Garcia compared the Fenton 2013 growth charts to those from the International Fetal and Newborn Growth Consortium for the 21st Century (Intergrowth 21st) Project (IW-21) for assessing to intra-(IUGR) and extrauterine (EUGR) growth restriction in very low birthweight infants. There was concordance between the charts for IUGR growth trajectories but not for EUGR infants. The dynamic IW-21 was more restrictive but better predicted morbidities in EUGR infants.

Reports on the negative effects of preterm birth included identifying predictors of severe necrotizing enterocolitis (NEC) and impaired neurodevelopmental outcomes and indicated that early identification of risk factors might ultimately lead to improved outcomes. Lin et al. sought to identify predictive indicators of necrotizing enterocolitis (NEC) by comparing biomarkers between NEC with portal venous gas (PVG) and NEC without PVG. C-reactive protein (CRP), fibrinogen degradation product, and blood glucose demonstrated predictive value for NEC-PVG. Current outcomes of the Neuroprem 2 cohort study were reported by Lugli et al. Out of 502 very preterm infants 9.6% had severe disability and 5.4% had cerebral palsy. Gestational age and periventricular hemorrhage were most highly associated with severe disability. Along these same lines, Wibowo et al. measured bone mineral content in newborns and found it lower in underdeveloped countries, higher in males, and negatively correlated with maternal cigarette usage. Hole et al. utilized a preterm piglet model to study early motor development. Preterm piglets took shorter steps than term piglets in early stages of walking but rapidly adapted with no differences within 3 days. Overall conclusions from the combined studies were that early interventions are needed to prevent later delays.

Khasawneh et al. retrospectively analyzed determinates of late hospital discharge. The majority of all preterm births analyzed were late preterm infants. While several parameters such as gestational age and maternal and neonatal morbidities were correlated with length of stay (early, ≤3 days vs. late >3 days), there was no correlation between length of stay after birth and later readmission.

In summary, growth restriction and small birth weight can have permanent consequences on the metabolic health of the infant. Furthermore, the morbidities associated with preterm birth such as NEC, and motor and neurodevelopmental disabilities are likely to contribute to deficits in the quality of life once these infants reach adulthood. Prematurity and SGA should be considered overall risk factors for adult health and quality of life.
